# On how correlations between excitatory and inhibitory synaptic inputs maximize the information rate of neuronal firing

**DOI:** 10.3389/fncom.2014.00059

**Published:** 2014-06-06

**Authors:** Pavel A. Puzerey, Roberto F. Galán

**Affiliations:** Department of Neurosciences, School of Medicine, Case Western Reserve UniversityCleveland, OH, USA

**Keywords:** stochastic Hodgkin-Huxley model, synaptic kinetics, input correlation, information, feed-forward inhibition

## Abstract

Cortical neurons receive barrages of excitatory and inhibitory inputs which are not independent, as network structure and synaptic kinetics impose statistical correlations. Experiments *in vitro* and *in vivo* have demonstrated correlations between inhibitory and excitatory synaptic inputs in which inhibition lags behind excitation in cortical neurons. This delay arises in feed-forward inhibition (FFI) circuits and ensures that coincident excitation and inhibition do not preclude neuronal firing. Conversely, inhibition that is too delayed broadens neuronal integration times, thereby diminishing spike-time precision and increasing the firing frequency. This led us to hypothesize that the correlation between excitatory and inhibitory synaptic inputs modulates the encoding of information of neural spike trains. We tested this hypothesis by investigating the effect of such correlations on the information rate (IR) of spike trains using the Hodgkin-Huxley model in which both synaptic and membrane conductances are stochastic. We investigated two different synaptic input regimes: balanced synaptic conductances and balanced currents. Our results show that correlations arising from the synaptic kinetics, τ, and millisecond lags, δ, of inhibition relative to excitation strongly affect the IR of spike trains. In the regime of balanced synaptic currents, for short time lags (δ ~ 1 ms) there is an optimal τ that maximizes the IR of the postsynaptic spike train. Given the short time scales for monosynaptic inhibitory lags and synaptic decay kinetics reported in cortical neurons under physiological contexts, we propose that FFI in cortical circuits is poised to maximize the rate of information transfer between cortical neurons. Our results also provide a possible explanation for how certain drugs and genetic mutations affecting the synaptic kinetics can deteriorate information processing in the brain.

## Introduction

The rate and timing of firing in cortical neurons is strongly affected by the interaction between synaptic excitation and inhibition (Salinas and Sejnowski, 2000). The architecture of cortical circuits ensures that the magnitude of excitatory and inhibitory synaptic inputs is approximately balanced on average and temporally correlated (Shu et al., 2003b; Haider et al., 2006), albeit with a small time delay for inhibition of ~1–10 ms (Wehr and Zador, 2003; Okun and Lampl, 2008; Wu et al., 2008). This correlation in amplitude and timing presumably arises in feed-forward inhibition (FFI) circuits, an anatomical motif present ubiquitously throughout the cortex which drives monosynaptic excitation and disynaptic inhibition onto target neurons (Porter et al., 2001; Sun et al., 2006; Cruikshank et al., 2007). The functional consequences of the correlations imposed by such a layout are far-reaching, encompassing a range of functions such as gain modulation for rapidly fluctuating synaptic inputs (Salinas and Sejnowski, 2000; Chance et al., 2002; Shu et al., 2003a; Pouille et al., 2009), shaping of neuronal tuning properties and stimulus selectivity (Wehr and Zador, 2003; Marino et al., 2005; Wu et al., 2006), directing the propagation of activity by selectively gating firing in neuronal ensembles (Kremkow et al., 2010a,b), and creating “windows of integration” during which excitatory inputs can temporally summate to promote spike generation before being rapidly suppressed by inhibition (Pouille and Scanziani, 2001; Pouille et al., 2009). Furthermore, Marsalek and colleagues demonstrated that small differences in the timing between presynaptic excitatory and inhibitory inputs (i.e., input correlation) is directly correlated with temporal jitter in postsynaptic spikes, i.e., output precision (Marsalek et al., 1997). Since neurons may represent information through the precise timing of spikes (decharms and Merzenich, 1996; Dan et al., 1998; Strong et al., 1998; Liu et al., 2001; Nemenman et al., 2008), it stands to reason that the control of spike timing by correlated excitation and inhibition is likely to govern the transfer of information between cortical neurons. Previous investigations using realistic simulations of cortical neurons have shown that, indeed, balanced excitatory and inhibitory synaptic currents maximize both coding and metabolic efficiency of neuronal spikes (Sengupta et al., 2013). In that study, however, the excitatory and inhibitory synaptic conductances were *uncorrelated* and as a result did not exhibit the correlation characteristic of cortical dynamics under experimental contexts (Wehr and Zador, 2003; Okun and Lampl, 2008; Wu et al., 2008). Furthermore, Kawaguchi and colleagues have shown that the relative balance between excitation and inhibition of a random synaptic input to simulated pyramidal neurons controls the maximal information content of spike trains in the presence of background synaptic noise (Kawaguchi et al., 2011), yet the timing of excitation and inhibition with respect to each other were not considered. To our best knowledge, the relevance of statistical correlations between balanced excitatory and inhibitory synaptic inputs for the information rates of neural spike trains have not been investigated. This may in part be due to the high computational cost of realistic simulations of stochastic neuronal dynamics. Here, we have overcome this limitation by using the stochastic-shielding approximation, which was recently introduced by our lab, accelerating stochastic simulations by up to two orders of magnitude while preserving accuracy (Schmandt and Galán, 2012).

A critical factor that influences the correlation between synaptic conductances and their effect on firing of cortical neurons is the time-course of the conductance change (Svirskis and Rinzel, 2000). This time-varying conductance shapes the trajectory of the membrane potential toward spike threshold and, consequently, alters the probability of firing an action potential. In support of this notion, previous findings have shown that the precision of spike-timing in pyramidal neurons has an inverse relationship with the decay kinetics of excitatory postsynaptic currents (Rodriguez-Molina et al., 2007), that is, spike time precision decrements as the postsynaptic currents slow down. The kinetics of postsynaptic synaptic responses may be modified by electrotonic filtering of the inputs across the dendritic arbor (Kleppe and Robinson, 1999), activation of distinct afferents (Walker et al., 2002), changes in the driving force (Salin and Prince, 1996), developmental changes in postsynaptic receptor (or receptor subunit) expression (Kirson and Yaari, 1996; Cohen et al., 2000; Bannister et al., 2005), interaction with intrinsic conductances (Miller et al., 1985; Wilson, 1995), and the presence of receptor-specific drugs (Orser et al., 1994; Poncer et al., 1996; Cohen et al., 2000). To our knowledge, the relationship between the kinetics of synaptic conductances and the information rates of neural spike trains has not been investigated.

In this study, we test the hypothesis that the rate of information transfer in cortical neurons depends on the correlation between concurrent excitatory and inhibitory synaptic inputs. We predict that an optimal time lag (δ) between excitation and inhibition would maximize information transfer between cortical neurons, since lags that are too short would preclude neuronal firing while long lags will likely decrease the precision of neuronal firing by prolonging the window of integration of presynaptic inputs. This prediction is consistent with a previous study showing an optimal time scale of rapidly fluctuating inputs for spike time reliability (Galán et al., 2008). Extending this hypothesis further, we predict that the rate of information transfer will depend on the kinetics of the synaptic conductance change, which inarguably affects the temporal correlation between synaptic excitation and inhibition (Svirskis and Rinzel, 2000). To test this hypothesis, we employ a biologically inspired Hodgkin-Huxley-type simulated neuron with stochastic ion channel gating (Schmandt and Galán, 2012) and drive it with Poisson trains of matched excitatory and inhibitory synaptic inputs. We test the impact of relative lag times between excitation and inhibition on the information rates of our model neuron across a range of lags and decay kinetics of synaptic conductances. Moreover, we compare the dependency of the information rates on the lags and kinetics in two synaptic regimes of (1) balanced conductances; and (2) balanced currents; this distinction is functionally important since the driving force can directly control the ratio between excitatory and inhibitory synaptic currents. Our findings reveal that the information rate (IR) of the neural spike train is indeed dependent on the synaptic kinetics as well as the relative delay times between excitation and inhibition. We show that the dependence of the IR on the synaptic kinetics shows an optimum at short and physiologically relevant monosynaptic delay times and that this dependence is present in the balanced currents, but not in the balanced conductances regime.

## Results

To investigate the role of temporal and cross-correlations in synaptic inputs on information transmission in cortical neurons we modeled excitatory and inhibitory synaptic conductances as two separate input channels injected into a single-compartment conductance-based Hodgkin-Huxley model neuron with stochastic biophysics. Ion channel stochasticity is essential for this model to recreate biologically faithful spike behavior (Fitzhugh, 1965; Skaugen and Walloe, 1979; Strassberg and Defelice, 1993; Schneidman et al., 1998). To carry out this computationally expensive task, we applied the stochastic shielding approximation (SSA) to ion channel gating, which has been shown to recreate the behavior of stochastic Hodgkin-Huxley models using substantially less computational power than other approaches while preserving accuracy (Schmandt and Galán, 2012). Central to our method was our ability to generate trains of synaptic inputs with Poisson statistics and precisely controlled temporal and cross-correlations. The temporal correlations of the barrages arise from the synaptic kinetics whereas cross-correlations are created by shifting two identical barrages relative to each other.

### Magnitude, kinetics, and correlation of synaptic excitation and inhibition

**Figure 1:**
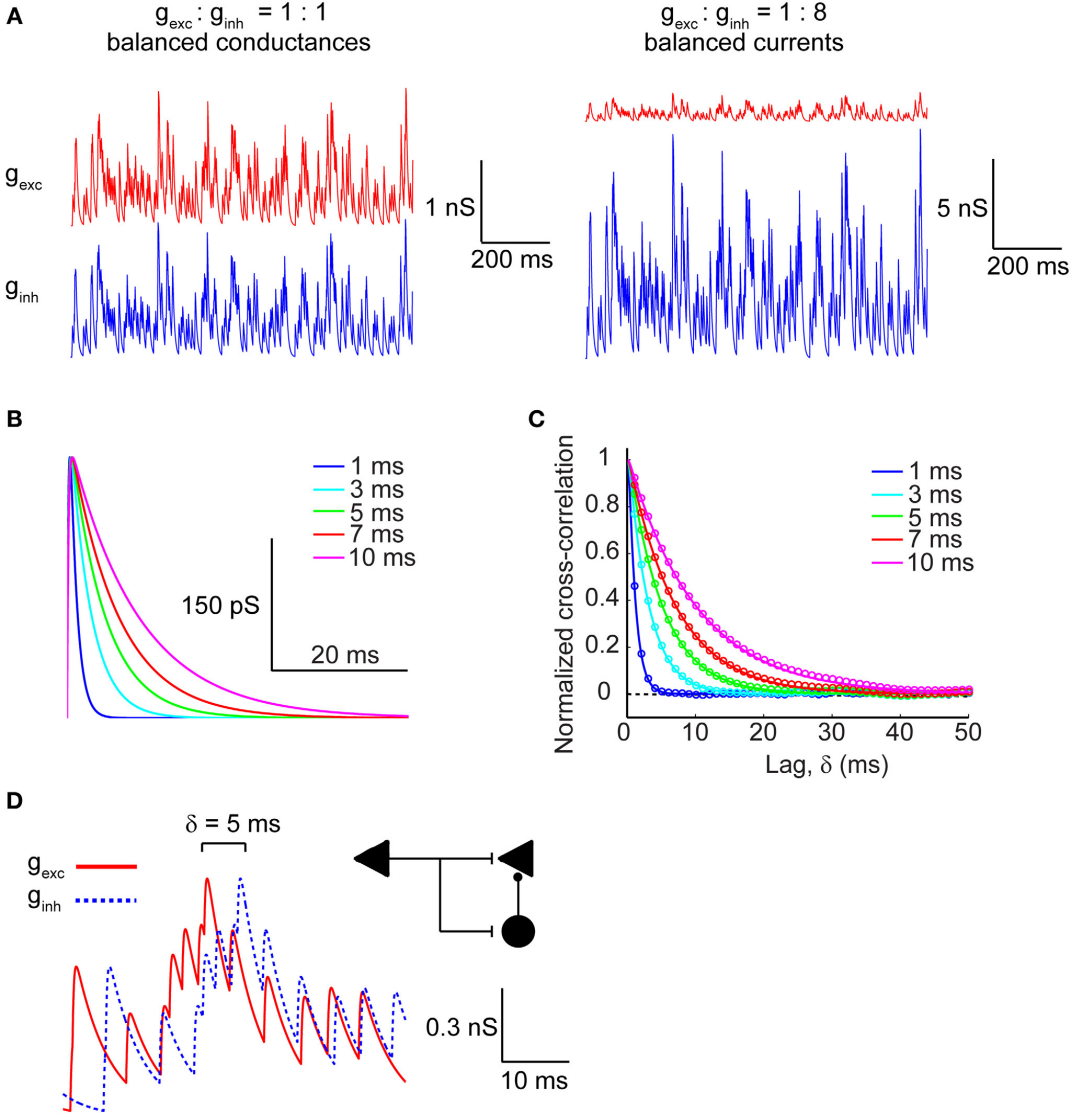
**Modeling excitatory and inhibitory synaptic inputs**. (**A**, left) Raw traces of identical and balanced excitatory (*g_exc_*; red trace) and inhibitory (*g_inh_*; blue trace) synaptic conductances. (**A**, right) Raw traces of excitatory and inhibitory synaptic conductances necessary to generate approximately balanced postsynaptic currents. Note that the vertical scale bars differ in magnitude between (**A**, left) and (**A**, right). **(B)** Raw traces of a single synaptic event plotted as the time course of the synaptic conductance for different values of τ, which corresponds to the decay time constant. **(C)** Cross-correlogram of balanced excitatory and inhibitory synaptic conductances shown in (**A**, left) calculated across a range of τ values. Colored circles correspond to the numerically determined cross-correlation of Poisson synaptic input trains, while solid colored lines correspond to the analytically derived cross-correlation (see Materials and Methods). Note that the width of the cross-correlation function broadens with increasing τ. **(D)** Raw traces of identical balanced excitatory and inhibitory synaptic conductances offset with respect to each other by δ = 5 ms. The inset shows a feed-forward inhibition circuit configuration that can generate such lags between identical trains of excitation and inhibition. The left triangle corresponds to the afferent input that activates excitatory target neurons (right triangle) monosynaptically and inhibitory interneurons (circle) disynaptically.

We modeled excitatory and inhibitory conductances as two separate channels of Poisson-distributed events whose rate was set by the fixed parameter λ (5 ms^−1^), which is inversely proportional to the average inter-event interval, and whose kinetics were varied across a range of τ values (1–10 ms), representing the time-constant of the synaptic conductance decay. This time constant introduces a temporal correlation (auto-correlation time) in the synaptic barrage (see Materials and Methods). Excitatory and inhibitory conductance amplitudes were either matched (*g_exc_* = 3 pS/μm^2^, *g_inh_* = 3 pS/μm^2^) to simulate the balanced conductances regime or the inhibitory conductance was multiplied by a factor of 8, which in our model generated approximately balanced excitatory and inhibitory synaptic currents (*g_exc_* = 3 pS/μm^2^, *g_inh_* = 24 pS/μm^2^) on average. Figure 1A represents the raw conductance traces in both the balanced conductance (Figure 1A, left) and balanced currents regimes (Figure 1A, right). For determining the effect of synaptic kinetics on information rates in cortical neurons, we generated synaptic input trains with different decay kinetics. The τ value was identical for a given pair of excitatory and inhibitory conductances, but was varied across different simulations. Figure 1B depicts a unitary synaptic conductance across a range of τ values. This value visibly sets the width of the time-varying conductance without affecting the rise time or the time at peak amplitude (Figure 1B). To understand how the kinetics shape the correlation structure between synaptic inputs, we analyzed the cross-correlogram between excitatory and inhibitory synaptic conductances as a function of the synaptic kinetics (Figure 1C). Clearly, the cross-correlation between excitation and inhibition is affected by the kinetics of the synaptic conductances, which are identical for both channels. As expected, the cross-correlation decays proportionally with the decay of the conductance waveform itself. Indeed, the numerically determined cross-correlation of the synaptic input trains (circles) accurately fits the analytically derived cross-correlation values (lines), as calculated in Materials and Methods. Central to our goal is the ability to also manipulate the cross-correlation between synaptic excitation and inhibition. To this end, we introduce a relative lag, δ, which delays the inhibitory conductance with respect to the excitatory. Figure 1D shows the raw conductance traces with inhibition lagging excitation by δ = 5 ms. In the context of cortical networks, such lagged correlations can arise through the FFI circuit (Figure 1D; inset schematic). This motif enables disynaptic inhibition generated by local interneurons (black circle) to lag behind monosynaptic excitation (black triangle) with delays ranging from 1 to 10 ms (Wehr and Zador, 2003; Okun and Lampl, 2008; Wu et al., 2008) thus, providing cortical neurons with windows of integration whose width is determined by the relative lag between excitatory and inhibitory conductances and their decay kinetics.

### Spiking behavior of a stochastic hodgkin-huxley neuron in response to kinetically variant synaptic inputs

**Figure 2:**
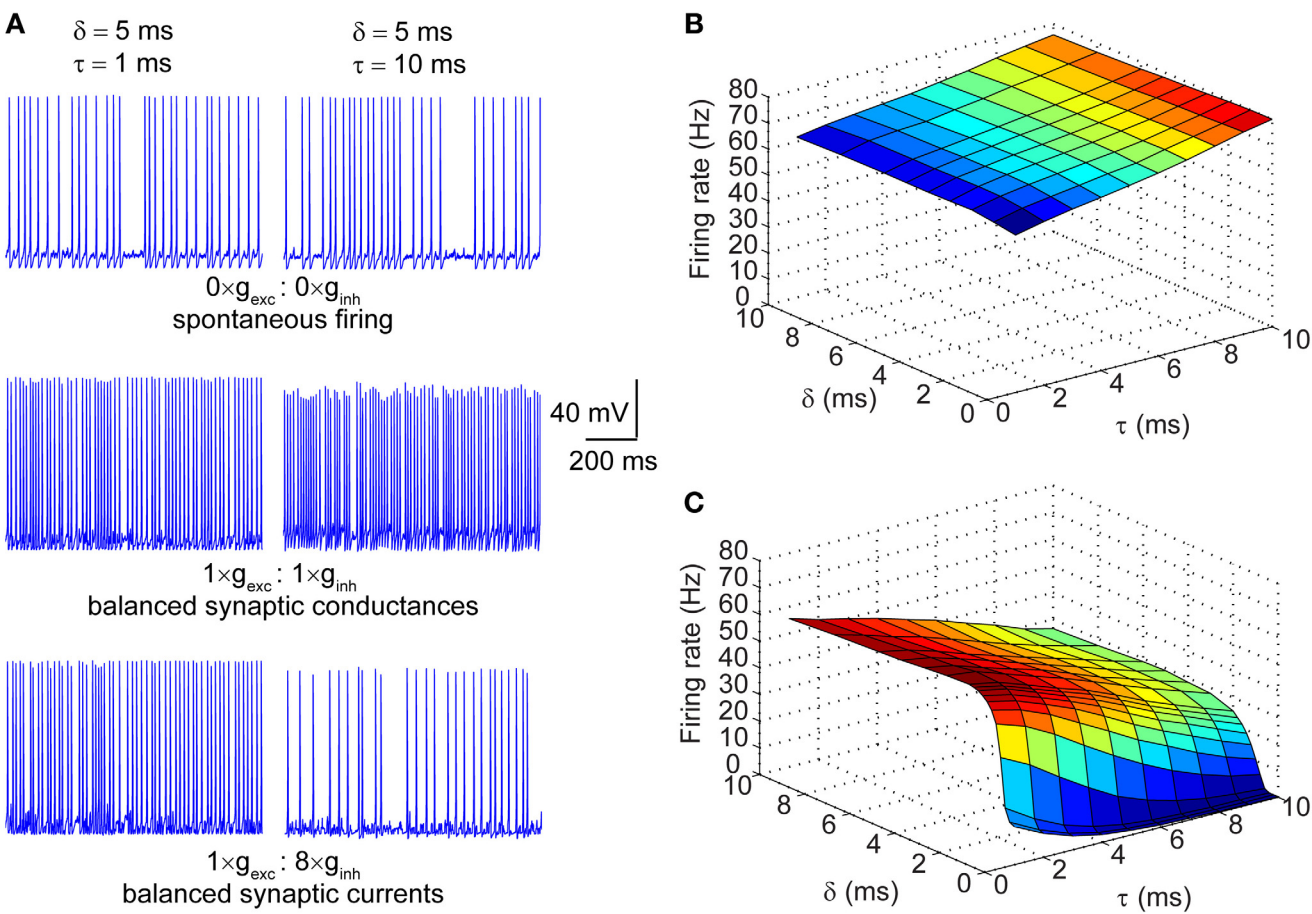
**Firing properties of a stochastic Hodgkin-Huxley neuron in different input regimes. (A)** Raw traces of membrane potential dynamics in three different input regimes: top row corresponds to spontaneous firing in the absence of synaptic inputs; middle row corresponds to firing in response to balanced synaptic conductances; bottom row corresponds to firing in response to balanced synaptic currents. The left column represents neuronal firing in response to synaptic events with very fast kinetics (τ = 1 ms) and the right column represents firing in response to synaptic events with slow kinetics (τ = 10 ms). Note that the offset between excitation and inhibition in all traces is set at δ = 5 ms. **(B)** Surface plot of firing rates of the model neuron in response to *balanced synaptic conductances* with varying synaptic kinetics (τ) and relative lags between excitation and inhibition (δ). **(C)** Surface plot of firing rates of the model neuron in response to *balanced synaptic currents* with varying synaptic kinetics and relative lags between excitation and inhibition.

The synaptic conductances described in the section above were injected into a single compartment Hodgkin-Huxley model of a neuronal membrane (100 μm^2^) with stochastic voltage-gated Na^+^ and K^+^ conductances, and a deterministic leak conductance. When the magnitude of the synaptic conductances was set to zero, the neuron fired spontaneously at ~30 Hz. This spontaneous firing resulted from the stochastic flickering of voltage-gated ion channels, as did the subthreshold oscillations of the membrane voltage seen during periods of quiescence (Figure 2A; top row). Depicted in Figure 2A are also the spike traces of the model neuron injected with matched excitatory and inhibitory conductances (middle row) and matched currents (bottom row). In both regimes, neurons were presented with trains of synaptic events with either fast (τ = 1 ms; left column) or slow kinetics (τ = 10 ms; right column). In the presence of excitatory and inhibitory *balanced conductances* with fast kinetics, the firing rate increased to ~60 Hz (Figure 2A; left column, middle row) and further increased to ~80 Hz when the kinetics were slow (Figure 2A; right column, middle row). In the synaptic input regime of balanced currents, the spike rate increased to ~60 Hz when the synaptic kinetics were fast, but dropped to ~30 Hz when the conductance decay was slow (Figure 2A; right column, bottom row). Thus, the decay time constant of the synaptic conductance impacts the firing rate differentially in the presence of balanced conductances vs. balanced currents. This becomes apparent when the firing rate is determined across the full range of synaptic kinetics and lags in both the balanced conductance (Figure 2B) and balanced currents (Figure 2C) regimes. Longer decay kinetics effectively increase the firing rate when the synaptic conductances are balanced and reduce the firing rate when the currents are balanced. Moreover, when the synaptic conductances are balanced, the firing rate shows no dependence on the lag between excitation and inhibition, but when the currents are balanced the firing rate is highly sensitive to short lags (0 > δ > 3 ms; Figure 2C).

### Entropy of neural spike trains

**Figure 3:**
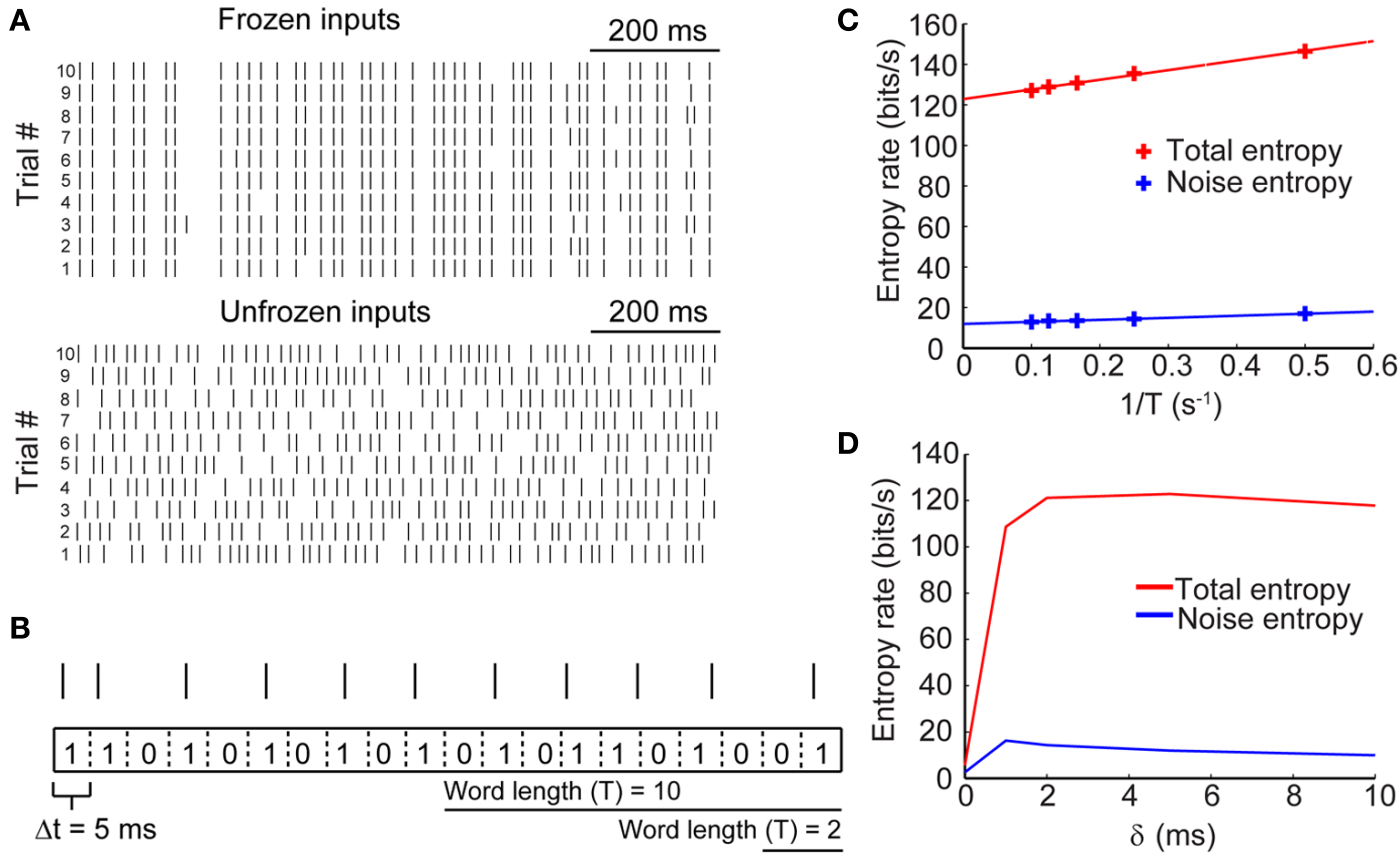
**Entropy of neural spike trains**. (**A**, top) Sample raster plots of neuronal firing in response to the presentation of a fixed stimulus (i.e., frozen input) across 10 trials. (**A**, bottom) Raster plots of neuronal firing in response to the presentation of different stimuli (i.e., unfrozen input) across 10 trials. **(B)** Schematic showing how spike trains (represented by spike raster) were converted to binary strings of 0's and 1's by binning the voltage trace into time bins of size Δ*t* = 5 ms. From these strings, words of various lengths were generated and the probability of their occurrence was calculated to yield entropy rates. **(C)** Entropy rates for spike responses in response to frozen input (noise entropy) and in response to unfrozen input (total entropy) calculated across different word durations. The true entropy rates were extrapolated by taking the entropy rate in the limit of T → ∞ (or 1/*T* → 0). **(D)** True entropy rates of neural spike trains in response to synaptic inputs with different lag times (δ) between excitation and inhibition.

Information content of a sequence of action potentials is by definition related to the variability of spike timing in response to an input signal. Repeated presentation of the same input conductance to the model neuron, therefore, enables us to measure the reproducibility of the resulting spike pattern. The top panel of Figure 3A shows the spike trains as in response to the same input conductance across ten trials (frozen input). Applying entropy measures as a proxy for spike variability, we obtain the “noise entropy” of the response across repeated presentations of the input signal. Noise entropy, however, informs us only about the spike variability to a single input pattern. To account for the full spectrum of potential spike responses of the model neuron, we presented a different set of input conductances across trials (unfrozen input; Figure 3A, bottom), this time yielding the “total entropy” of the spike train. Entropy measurements were carried out using the “direct method” (see Materials and Methods), which involved converting the output signal into a binary string of 0's and 1's by binning the spike trace with small time windows (Δ*t* = 5 ms) and counting spikes within each bin. A bin containing no spikes corresponds to a 0, while a bin containing one or more spikes corresponds to a 1 (Figure 3B). We then generated sequences of words of various lengths (T = Δ*t*× number of bins) which were then used to calculate the entropy based on the probability of occurrence of each possible word. Entropy, being an extensive property, scales with the length of the signal being measured and is sensitive to the temporal resolution of binning (Strong et al., 1998). Thus, to estimate the maximal entropy of the spike trains we extrapolated the entropy for words of length T → ∞ (Figure 3C). Entropies were normalized by time to give entropy rates per time unit (bits/s).

We calculated the entropy rates for sets of paired excitatory and inhibitory conductances across a range of time lags for inhibition. Figure 3D shows the entropy rates as a function of δ for the sample traces shown in Figure 3A. These rates are exemplary of only a single value of the synaptic kinetics and are presented strictly heuristically. Our results show that the total and noise entropies are initially very low when excitatory and inhibitory conductances occur simultaneously but rise rapidly across a short range of δ values until they plateau around δ = 2 ms. The following sections will deal with the use of these entropy rates for the determination of spike train information rates.

### Information rate of spike trains is insensitive to synaptic kinetics and the relative delay of synaptic inhibition in the balanced conductances regime

**Figure 4:**
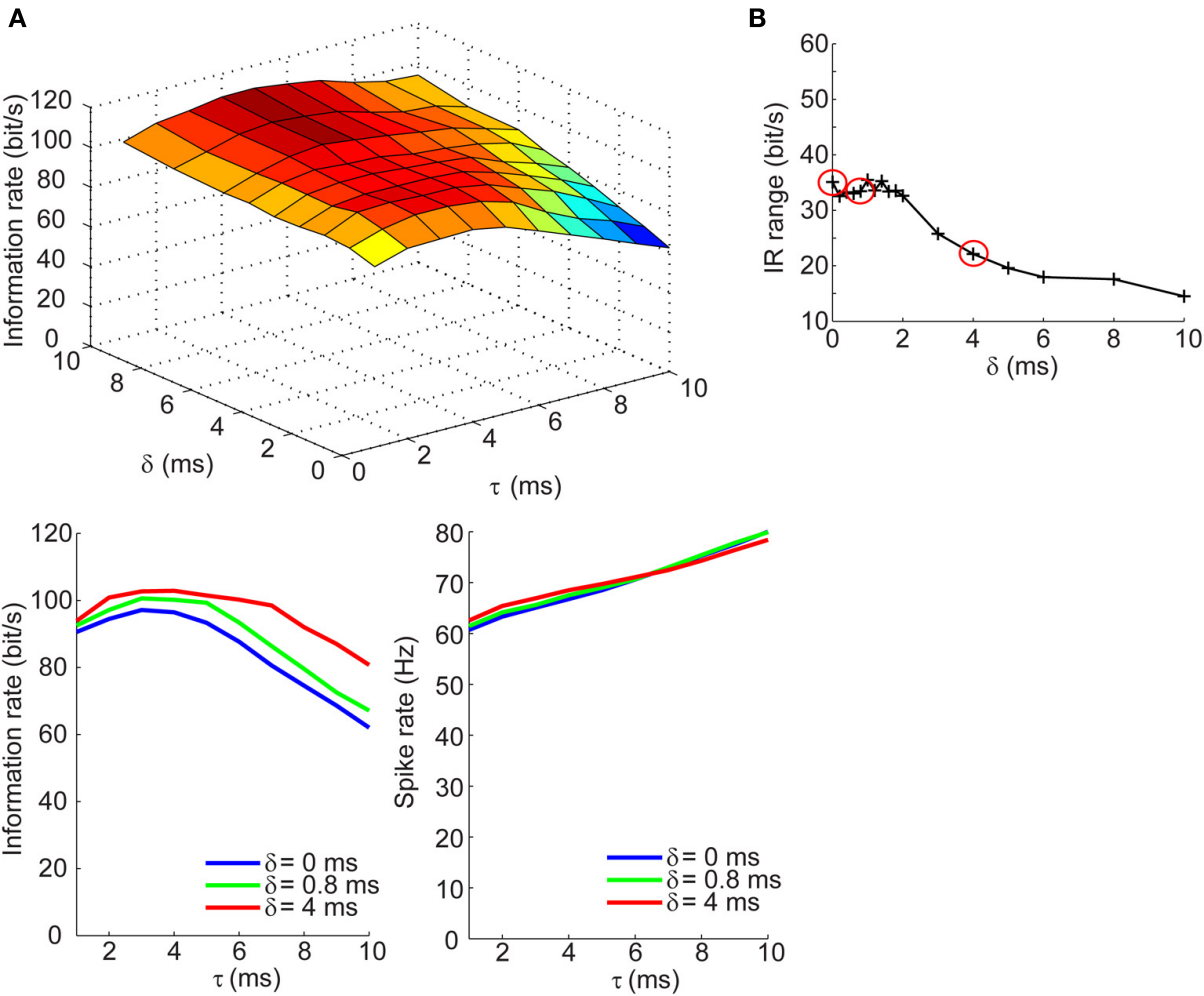
**Information rates of neural spike trains in the balanced conductances regime**. (**A**, top) Surface plot of the information rate of neural spike trains as a function of the synaptic kinetics (τ) and delays in inhibition relative to excitation (δ). (**A**, bottom left) Information rate as a function of synaptic kinetics for three different values of δ. This plot corresponds to three different slices taken from (**A**, top). (**A**, bottom right) Plot of firing rate as a function of synaptic kinetics for the same three δ values presented in (**A**, bottom left) shows that the dependency of the information rate on the synaptic kinetics is not accounted for by similar changes in firing rate. **(B)** Range of information rate as a function of relative delay between excitation and inhibition. The red dots correspond to the three values of δ shown in (**A**, bottom left) and (**A**, bottom right) (see Results for explanation).

Measuring the information rates of a neural spike trains requires that we take the difference between the total and noise entropy rates. This difference quantifies the IR without necessitating assumptions about the nature of the signal being represented. We applied this measure to spike trains generated across a range of τ and δ values to assess the dependence of the IR on the temporal correlation of excitatory and inhibitory synaptic inputs. This was first done for the balanced conductances regime. The top panel of Figure 4A shows the dependence of the information across a range of τ and δ values. The IR remains high and constant across different values of δ when the synaptic kinetics are fast (τ < 4 ms) and increases by no more than 40% with increasing lags when the kinetics are slow (τ > 5 ms). The bottom left panel of Figure 4A shows three slices taken from the surface plot corresponding to the IR for three values of δ as a function of the kinetics. The selection of these three points will be clearly explained in the next section. Visible from this panel is that for the three different δ values, the change in IR follows a similar trajectory: for fast kinetics the IR slowly increases until reaching a maximum at ~3 ms and then decreases for higher values of τ. The relationship between the IR and τ cannot be accounted for by changes in firing rate, which increases monotonically with increasing τ for all values of δ (Figure 4A, bottom right; Figure 2B). To quantify the range of information rates of the spike train across the full range of synaptic kinetics, we took the difference between the maximal and minimal IR values along the τ dimension for different δ values and saw that the IR range was highest for short lags (35 bits/s) and decreased steadily with increasing lags. This decrease in the IR range of the spike train corresponds to the flattening of the IR curve across the τ dimension with increasing values of δ.

### Information rate of spike trains exhibits dependence on synaptic kinetics at short delays for inhibition in the balanced currents regime

**Figure 5:**
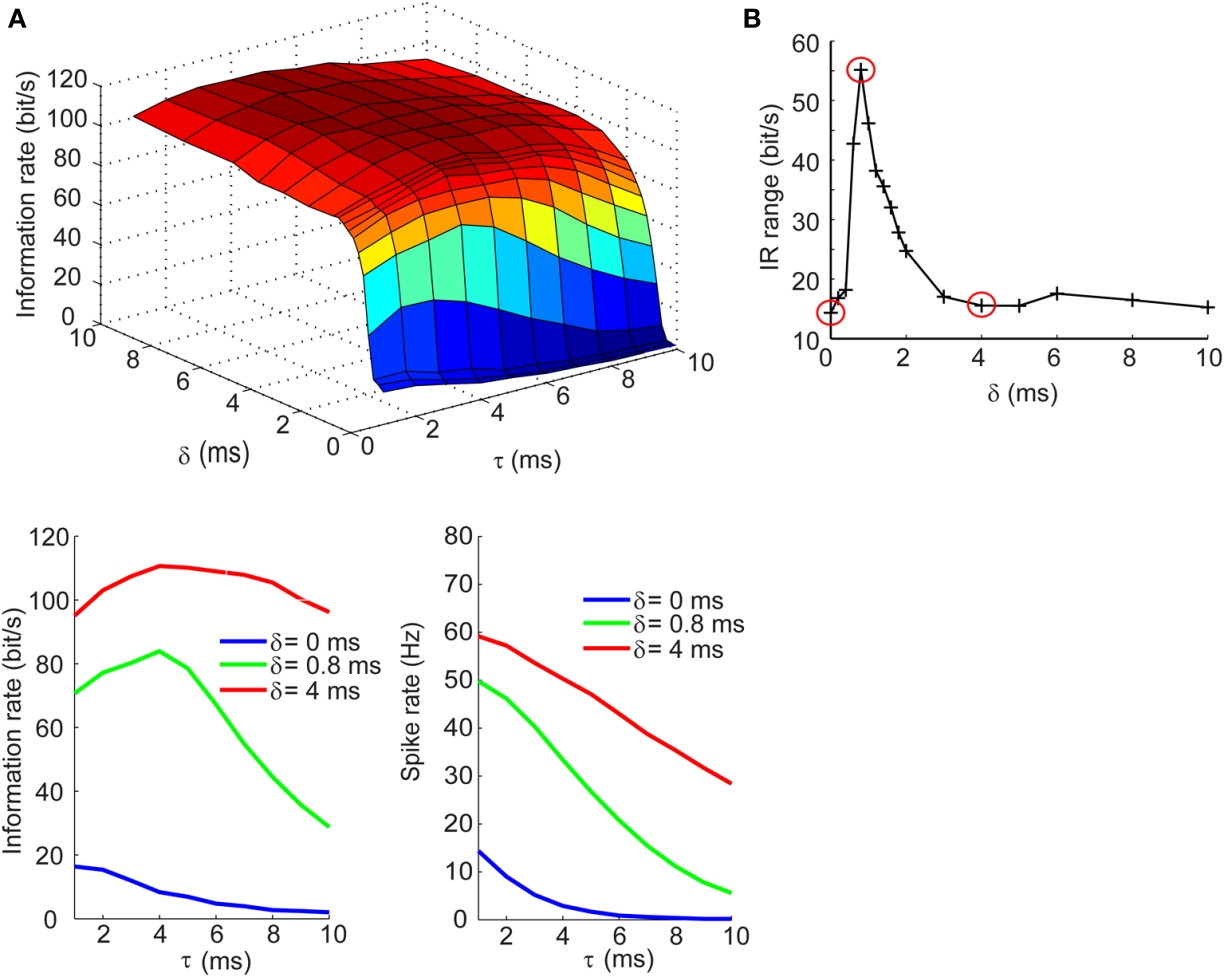
**Information rates of neural spike trains in the balanced currents regime**. (**A**, top) Surface plot of the information rate of neural spike trains as a function of the synaptic kinetics (τ) and delays in inhibition relative to excitation (δ). (**A**, bottom left) Information rate as a function of synaptic kinetics for three different values of δ. This plot corresponds to three different slices taken from (**A**, top). (**A**, bottom right) Plot of firing rate as a function of synaptic kinetics for the same three δ values presented in (**A**, bottom left) shows that the dependency of the information rate on the synaptic kinetics is not accounted for by similar changes in firing rate. **(B)** Range of information rate as a function of relative delay between excitation and inhibition. The red dots correspond to the three values of δ shown in (**A**, bottom left) and (**A**, bottom left). Note the three points circled in red correspond to the peak IR range values and two non-adjacent δ that do not exhibit the optimum in information rate as a function of kinetics.

We next determined how the IR changes with τ and δ in the balanced synaptic currents regime. The surface plot in top panel of Figure 5A shows an entirely different dependency of the IR on synaptic kinetics and relative lags times. For δ > 2 ms, the IR remains high and relatively constant across different values of τ; however, as the synaptic lags decrease below 2 ms, the IR begins to show an optimal dependence to the synaptic kinetics. The bottom left panel of Figure 5A shows the IR as a function of τ for three values of δ. These results show that for instantaneous lags (δ = 0 ms) the IR is relatively low (*IR* < 20 bits/s) and decreases slowly across the τ dimension; for δ = 4 ms, the IR does not undergo dramatic changes and remains relatively high (*IR* > 95 bits/s); for δ = 0.8 ms, however, the IR begins at an intermediate value (70 bits/s) and increases until it reaches an optimum at τ = 4 ms, then drops 55% relative to the maximal value (Figure 5A, bottom left). Again, this dependency of the IR on the kinetics cannot be explained by changes in firing rate which decrease approximately monotonically with increasing τ values (Figure 5A, bottom right). Applying the same analysis used in the previous section, we compute the IR range across the τ dimension for different values of δ and observe that the IR exhibits the most dramatic dependence of synaptic kinetics at an optimal value of the relative lag (δ = 0.8 ms). Thus, the selection of the three δ values shown in the bottom panels of Figures 4A, 5A and circled in red in Figures 4B, 5B are based on the range of δ values within which the optimum occurs (δ = 0 ms: δ = 4 ms) and the δ value at the optimum (δ = 0.8 ms). This optimal dependence of the IR on synaptic kinetics is only present when the synaptic currents are balanced, but not the synaptic conductances (Figures 4B, 5B). Incidentally, when the synaptic input trains are normalized by the integral of their conductance, the IR decreases slowly and monotonically with τ in the balanced conductance regime and the observed peak of the IR as a function of τ disappears in the balanced currents regime (data not shown). This normalization, however, is not physiologically relevant considering that changes in synaptic kinetics in biological neurons are not compensated for by changes in the amplitude of the synaptic inputs on an event-by-event basis.

## Discussion

In this study we set out to investigate how the encoding of information in neurons depends on the temporal and cross-correlation of balanced synaptic inputs. We manipulated the correlation between identical trains of excitatory and inhibitory inputs by directly controlling the decay kinetics (τ) of the synaptic conductance and/or the relative time delay between excitation and inhibition (δ), with inhibition always lagging behind. Our results show that the encoding of information in neural spike trains exhibits a dependence on the correlation between balanced excitatory and inhibitory synaptic currents and that this dependence is absent in the input regime of balanced synaptic conductances. Specifically, findings reported herein demonstrate that the synaptic kinetics modulate the IR range at which the spike train maximally encodes information, but do so only when synaptic inhibition lags behind excitation with very short monosynaptic delays (δ < 2 ms). Furthermore, our model exhibits an optimal delay (δ = 0.8 ms) for inhibition at which the modulation of the IR by the synaptic kinetics is highest. Such delays between excitation and inhibition are within the physiological range of monosynaptic lags obtained from *in vitro* and *in vivo* recordings of synaptic barrages in cortical neurons (Wehr and Zador, 2003; Okun and Lampl, 2008; Wu et al., 2008). The optimum of the IR as a function of τ emerges as the result of the following: As stated in the Materials and Methods, the IR is determined as a difference between the total and noise entropies which represent the variability of the spike patterns in response to unfrozen and frozen input trains, respectively. Intuitively and empirically, the total entropy is substantially larger across different values of τ and δ and changes most drastically at values of δ < 2 ms (data not shown), at which the neuronal spiking is subject to a dramatic modulation by the inhibitory inputs. It is at this exact range of δ that the noise entropy is the highest across the τ (for τ < 5 ms) and δ dimensions. Why is the noise entropy highest when synaptic kinetics are fast? To answer this question we consider the relationship between synaptic input-driven spiking and spontaneous firing from stochastic fluctuations of intrinsic regenerative conductances. During synaptic bombardment, the synaptic conductance is the dominant driver of neuronal firing as it overwhelms the intrinsic conductances in both magnitude and duration. However, with increasing synaptic kinetics (smaller τ), the integral of the synaptic conductance decreases and the dominance of the synaptic conductance abates, so that stochastic fluctuations of the intrinsic conductances allow for spontaneous firing. As a result, the spike patterns become more variable, thereby increasing the noise entropy. Thus, the peak of the IR emerges as a result of this increase in the noise entropy at small values of τ and δ and endows the neuronal membrane with the observed dependence of the IR on synaptic kinetics. These results are consistent with a previous report showing that spike-time reliability, an analytic measure related to the information capacity of a spike train, shows an optimal value at specific auto-correlation times of their uncorrelated synaptic inputs (Galán et al., 2008). Therefore, we conclude that synaptic kinetics as well as relative delays between synaptic excitation and inhibition may be tuned to optimize information transfer between neurons.

The time-course of the postsynaptic response may be subject to modulation by various factors including electrotonic distance of inputs from sites of integration (Kleppe and Robinson, 1999), pattern of afferent activation (Walker et al., 2002), driving force (Salin and Prince, 1996), postsynaptic receptor (and subunit) type (Kirson and Yaari, 1996; Cohen et al., 2000; Bannister et al., 2005), intrinsic conductances (Miller et al., 1985; Wilson, 1995), and the presence of receptor-specific drugs (Orser et al., 1994; Poncer et al., 1996; Cohen et al., 2000). The many ways in which the kinetics of the postsynaptic response to incoming inputs can be altered provides cortical neurons with a myriad of mechanisms to tune the correlation structure of incoming synaptic inputs. In particular, drugs, neuromodulators, etc. may change the synaptic kinetics to the point that the IR is outside its range, thereby deteriorating the processing of information in the brain and altering the state of awareness and consciousness.

We have shown here that the IR is also sensitive to the arrival times of inhibition with respect to excitation. Precisely controlling monosynaptic delay times for inhibition may be less trivial than tuning the synaptic kinetics, but is still possible. In the context of a feed-forward inhibitory circuit, one potential mechanism to tune inhibitory lags may be to alter the integration times of the feed-forward interneuron. Experiments in rats have shown that integration time in layer 4 stellate cells of somatosensory cortex is tightly regulated by thalamocortical FFI, thus controlling the precise spike timing of those neurons (Gabernet et al., 2005). Cortical interneurons also receive reciprocal inhibition (Lee et al., 2013; Pfeffer et al., 2013; Pi et al., 2013) and, as a consequence, are likely to have their integration windows regulated by inhibitory circuits. The size of the integration window of the feed-forward interneuron would control its precise spike timing and resultantly the lag of the inhibition in the excitatory neuron. Another way in which delays in inhibition can be modulated in a feed-forward circuit is through recruitment of distinct inhibitory networks (Beierlein et al., 2003). These networks are comprised of molecularly and physiologically distinct interneuron populations that exhibit differential responsiveness to temporally patterned inputs and distinct synaptic dynamics.

Pivotal to the simulations carried out in this study was our ability to efficiently simulate the spike behavior of the model neuron across numerous trials (sampling rate = 10 KHz; 5 s/trial; 56 trials for each δ and τ; which yields ~2.2 Gigabytes per data point in Figures 4A, 5A). Stochastic simulations of ion channels are notoriously expensive computationally and often create the bottleneck for generating sufficient data across a large enough parameter range. We used the SSA for simulating stochastic ion channel gating dynamics (Schmandt and Galán, 2012) to avoid this problem. The SSA reduces the number of ion channel states requiring stochastic simulation, and therefore, dramatically reduces the computational load.

Modeling of the synaptic inputs required that several assumptions be made about the nature of cortical excitation and inhibition. First, the model assumes that excitatory and inhibitory synaptic inputs are correlated. This assumption has been validated by *in vitro* (Graupner and Reyes, 2013) and *in vivo* (Wehr and Zador, 2003; Okun and Lampl, 2008; Wu et al., 2008) recordings of synaptic barrages from cortical neurons showing that, indeed, excitation and inhibition are correlated in magnitude and timing, with inhibition tracking excitation by a few milliseconds. Secondly, the rate of synaptic events in time was assumed to be fast (5 ms inter-event interval), corresponding to high levels of correlated activity in presynaptic neurons. Recordings from cortical neurons in awake behaving mice during sensory stimulation (Crochet and Petersen, 2006), in anaesthetized ferrets during spontaneous active states (Haider et al., 2006), and in spontaneous active cortical slices (Compte et al., 2008) have confirmed high rates of synaptic bombardment, therefore, lending validation to the use of high rates of synaptic activity in our model. It is important to note, however, that synaptic inputs onto cortical neurons have also been shown to occur as sparse and synchronous population events (Wehr and Zador, 2003; DeWeese and Zador, 2006). Our study focused exclusively on synaptic regimes with high levels of activity, thus, it will be important to understand how temporal correlations between excitation and inhibition in sparse regimes affect information encoding. Previous findings by Miura et al. suggest that balanced excitation and inhibition in cortical neurons may in fact decouple irregularity of the spike train from rate modulations in firing, which may arise from changes in the synaptic input rate (Miura et al., 2007). Thus, the IR of the spike trains, being dependent on irregularity of the spike times, may be insensitive to changes in synaptic input rate if excitation and inhibition are balanced. On a related note, the magnitude of synaptic inhibition has been shown to have an inverse relationship with the overall rate of synaptic activity (Taub et al., 2013). This dependence shifts the relative balance between excitation and inhibition and may have a profound effect on encoding of information in cortical neurons. Future studies will need to address this problem to better understand the role of synaptic dynamics in neural coding.

The bulk of our study focused on the role of balanced synaptic inputs in encoding of information. A previous study by Sengupta and colleagues demonstrated that uncorrelated and balanced synaptic currents maximize the coding and metabolic efficiency of neuronal spikes by reducing the spike rate without substantially affecting the information rates (Sengupta et al., 2013). Our work applies the information theoretic approach using a similar model of a stochastic Hodgkin-Huxley neuron to address a different question: Are the correlations between synaptic inputs relevant for information processing? Our results indeed show a dependency of information encoding on the correlation between balanced synaptic currents. Moreover, the dependence of the information rates on the synaptic kinetics cannot be accounted for by changes in firing rate. Though balanced synaptic currents effectively decrease the firing rate as the kinetics slow down, this relationship is monotonic and does not exhibit the optimum dependence to the kinetics seen for the IR.

Using a conductance-based single-compartment Hodgkin-Huxley model offers insight into the interaction between synaptic inputs and an active neuronal membrane, but it ignores the complex shape and electrotonic geometry of cortical neurons. These features are important for spatiotemporal integration of synaptic inputs (Bernander et al., 1991) since distal dendritic inputs may be processed differently due to interactions with active dendritic conductances (Miller et al., 1985), differences in electrotonic properties (Kleppe and Robinson, 1999) or longer integration times caused by differences in FFI (Pouille and Scanziani, 2001; Pouille et al., 2009). Future studies should consider the complex geometry of cortical neurons and how it may impact information processing.

An important aspect of the work presented herein is its focus on information encoding at the level of individual neurons. Though single cells certainly have the capacity to encode and represent information (Nemenman et al., 2008), distributed networks of anatomically and functionally connected neurons (i.e., neural ensembles) also carry out this task (Nicolelis et al., 1995; Rothschild et al., 2010; Ince et al., 2013). The role of balanced synaptic inputs on information transfer in cortical networks has been addressed in previous studies. For instance, using multi-site recordings of local field potentials (LFP) in rats and monkeys, Shew et al. showed that cortical networks with balanced excitation and inhibition maximize information capacity and transfer (Shew et al., 2011). Our results are in agreement with these findings, showing optimal information rates of neural spike trains when synaptic currents are balanced. It is important to note, however, that LFP recordings of cortical networks capture coordinated activity of large ensembles of neurons operating at substantially slower time-scales than that of single neurons. Thus, the nature of the computations performed and information encoded at the level of single cells vs. that of neural ensembles is likely to have marginal correspondence.

In conclusion, we provide a biologically realistic model of neurons with stochastic ion channel biophysics and synaptic inputs and apply information theoretic approaches to show that information rates of neural spike trains is dependent on the temporal correlation of balanced synaptic currents. Our findings emphasize the importance of these correlations for information encoding and suggest that cortical neurons may optimize this process through precise tuning of synaptic kinetics and timing of excitatory and inhibitory inputs.

## Materials and methods

### Synaptic inputs

All simulations were carried out using the Matlab R2013b software package (Mathworks). We modeled the synaptic events as Poisson trains with a rate of λ = 5 ms^−1^, which was fixed across all simulations. The synaptic train was then convolved with an “alpha function” to yield the time-dependent conductance, g(*t*), of the following form,

(1) $$g(t)=G\left(e^{-t/\tau_{r}}-e^{-t/\tau }\right)$$

where the constant *G* is set at 300 pS for all simulations trials in the balanced conductances regime, *e* is the base of the natural logarithm, and τ_*r*_ and τ are the rise and decay time-constants, respectively. τ_*r*_ is set at a fixed value of 0.2 ms while τ is varied across the range of 1 ≤ τ ≤ 10 ms. The excitatory and inhibitory synaptic conductances (*g_exc_* and *g_inh_*, respectively) were created as identical realizations of a Poisson process in the balanced conductances input regime. For the balanced currents input regime, the inhibitory conductance was multiplied by a factor of 8, yielding a maximal conductance amplitude of 2400 pS. To generate conductance traces in which inhibition lagged behind excitation, we offset the two waveforms by a lag time, δ, which for a given simulation was taken from a range of lags, (1 ≤ δ ≤ 10 ms).

### Analytical expression for the cross-correlogram of the synaptic inputs

To calculate an analytical expression for the cross-correlogram of the excitatory and inhibitory synaptic inputs, we first note that the inhibitory input is identical with the excitatory input but delayed with a lag, δ, so that the cross-correlogram *C*(δ) is actually equivalent to the auto-correlogram of the excitatory input. We also note that the excitatory input is the convolution of a Poisson process with the kinetics of a single synaptic event given by (1) and recall the following two theorems of time-series analysis: 1) The Wiener-Khinchin theorem, stating that for a given signal the auto-correlogram is the Fourier transform of its power spectrum; and 2) the convolution theorem, stating that the power spectrum of the convolution of two signals is the product of their power spectra. Therefore, since the power spectrum of a Poisson process is a constant, the cross-correlogram is determined by the Fourier transform of the power spectrum of a single synaptic event. Defining α = 1/τ_*r*_ and β = 1/τ, the power spectrum of a single synaptic event is given by:

(2) $$k{\left|\int_{0}^{\infty }\left(e^{-\alpha t}-e^{-\beta t}\right)\;e^{i\omega dt}\right|}^{2}=k\frac{{\left(\alpha -\beta \right)}^{2}}{\left(\alpha^{2}+\omega^{2}\right)\left(\beta^{2}+\omega^{2}\right)},$$

where *k* is a constant and |…|^2^ denotes the square of the modulus of a complex number. The un-normalized cross-correlogram of the excitatory and inhibitory inputs is then given by the Fourier transform of (2)

(3) $$C(\delta )=\frac{k}{2\pi }\int_{-\infty }^{\infty }\frac{{\left(\alpha -\beta \right)}^{2}}{\left(\alpha^{2}+\omega^{2}\right)\left(\beta^{2}+\omega^{2}\right)}\;e^{i\omega \delta }d\omega .$$

To solve the integral in (3) we apply the residues theorem to a closed integration path containing two poles on the upper-half of the complex plane, ω = *i*α, *i*β, whose respective residues are

$$i\frac{\left(\alpha -\beta \right)e^{-\alpha \delta }}{2\alpha \left(\alpha +\beta \right)}\;\text{and}\;-i\frac{\left(\alpha -\beta \right)e^{-\beta \delta }}{2\beta \left(\alpha +\beta \right)}.$$

Thus, expression (3) yields

$$C(\delta )=\frac{k\left(\alpha -\beta \right)}{2\left(\alpha +\beta \right)}\left(\frac{e^{-\beta \delta }}{\beta }-\frac{e^{-\alpha \delta }}{\alpha }\right).$$

Finally, the cross-correlogram, normalized so that *C* (0) = 1 reads

$$C(\delta )=\frac{\tau_{r}e^{-\delta /\tau_{r}}-\tau e^{-\delta /\tau }}{\tau_{r}-\tau }.$$

This analytical expression accurately describes the cross-correlogram obtained from the numerical simulations, as shown in Figure 1C.

### Single compartment model

Neuronal dynamics were simulated in a single compartment model of a Hodgkin-Huxley (Hodgkin and Huxley, 1952) neuron with stochastic voltage-gated fast Na^+^, delayed rectifier voltage-gated K^+^ channels, and a deterministic leak conductance as detailed in (Schmandt and Galán, 2012). Modeling of stochastic ion channel gating was made more computationally efficient by applying the SSA of Markov chains, which reduces observable states (Schmandt and Galán, 2012). The fluctuations in the membrane voltage were described by the following current balance equation:

$$\begin{array}{l} C_{m}\frac{dV}{dt}=\overset{\text{membrane}\;\text{currents}}{\overset{︷}{g_{Na}(t)\left(E_{Na}-V(t)\right)+g_{K}(t)\left(E_{K}-V(t)\right)+g_{leak}(E_{leak}-V)}}+\\ \;\;\;\;\;\;\;\;\;\;\;\;\;\;\;\;\;\;\;\;+\underset{\text{synaptic}\;\text{currents}}{\underset{︸}{g_{exc}(t)\left(E_{exc}-V(t)\right)+g_{inh}(t)\left(E_{inh}-V(t)\right)}} \end{array}$$

where *C_m_* corresponds to the membrane capacitance, *g_Na_*, *g_K_*, and *g_leak_* are the Na^+^, K^+^, and leak conductances with their respective reversal potentials, *E_Na_*, *E_K_*, and *E_leak_*. The excitatory and inhibitory synaptic conductances (*g_exc_* and *g_inh_*) along with their respective reversal potentials, *E_exc_* (0 mV) and *E_inh_* (−80 mV), dictate the extent to which synaptic currents affect the membrane potential fluctuations. The membrane potential was simulated with a time resolution of *dt* = 0.01 ms.

### Determination of information rates

Spike train entropy was determined using the “direct method” (Nemenman et al., 2008). This approach quantifies the entropy of the spike trains without making assumptions about the nature of the stimulus. Spike trains were binned in small time windows (Δ*t* = 5 ms) and spikes were counted for each bin. A value of 0 was assigned to each bin containing no spikes and a value of 1 for those containing one spike. With the maximal firing rate of the model peaking at 80 Hz (inter-spike interval = 12.5 ms), our choice of Δ*t* ensures that at most one spike can occur within a given time bin, therefore, providing information rates for timing of action potentials with millisecond precision. The resultant binary strings of 0's and 1's were used to generate words of length *n* where *n* = 2, 4, 6, 8, 10, yielding words that spanned time windows of *T* = *n*Δ*t*. Probability distributions were then generated to quantify the occurrence probability of a given word, *P(W)*, within a response pattern. Noise entropy, which measures the reproducibility of spike trains in response a fixed input stimulus across trials (56 trials), was measured as with respect to the conditional probability of a word occurring at time *t* and calculated with the following equation:

$$H_{noise}={\langle -\sum_{W}^{P}\left(W|t\right)\log_{2}P\left(W|t\right)\rangle }_{t}$$

where the operator 〈…〉_*t*_ denotes averaging over time. The total entropy, which quantifies the possible permutations of output patterns with respect to a broad set of inputs, was determined by presenting the model neuron with a different input pattern across 56 trials and measuring the occurrence probability of a given word. The total entropy was calculated as:

$$H_{total}=-\sum_{W}P(W)\log_{2}P(W).$$

By definition, the information encoded by the spike train is the difference between the total and noise entropies. We thus computed the information as:

(4) $$I=H_{total}-H_{noise}.$$

Both the noise and the total entropies were normalized by *T* to yield entropy rates (bits/s). Since entropy is sensitive to the word length, we extrapolated the entropy rates in the limit of *T* → ∞, yielding the true rates, and using (4) the IR.

## Author contributions

Pavel A. Puzerey and Roberto F. Galán conceived and designed the experiments. Pavel A. Puzerey performed the experiments. Pavel A. Puzerey and Roberto F. Galán carried out data analysis. Pavel A. Puzerey generated the figures. Pavel A. Puzerey and Roberto F. Galán wrote the manuscript.

### Conflict of interest statement

The authors declare that the research was conducted in the absence of any commercial or financial relationships that could be construed as a potential conflict of interest.
